# Survival analysis of patients with advanced-stage nasopharyngeal carcinoma according to the Epstein-Barr virus status

**DOI:** 10.18632/oncotarget.8144

**Published:** 2016-03-17

**Authors:** Hao Peng, Lei Chen, Yuan Zhang, Rui Guo, Wen-Fei Li, Yan-Ping Mao, Ling-Long Tan, Ying Sun, Fan Zhang, Li-Zhi Liu, Li Tian, Ai-Hua Lin, Jun Ma

**Affiliations:** ^1^Department of Radiation Oncology, Sun Yat-sen University Cancer Center, State Key Laboratory of Oncology in Southern China, Collaborative Innovation Center for Cancer Medicine, Guangzhou 510060, People's Republic of China; ^2^Imaging Diagnosis and Interventional Center, Sun Yat-sen University Cancer Center, State Key Laboratory of Oncology in Southern China, Collaborative Innovation Center for Cancer Medicine, Guangzhou 510060, People's Republic of China; ^3^Department of Medical Statistics and Epidemiology, School of Public Health, Sun Yat-sen University, Guangzhou 510275, People's Republic of China

**Keywords:** nasopharyngeal carcinoma, Epstein-Barr virus, prognosis, advanced stage, intensity-modulated radiation therapy

## Abstract

**Purpose:**

The main aim of this study is to analyze the prognostic differences in nasopharyngeal carcinoma (NPC) patients who are positive and negative for Epstein-Barr virus (EBV).

**Results:**

Of the 1106 patients, 248 (22.4%) had undetectable pre-treatment plasma EBV DNA levels. The total distant metastasis rate for EBV-negative group vs. EBV-positive group were 3.6% (9/248) vs. 15.0% (128/858) (*P* < 0.001). The estimated 4-year disease-free survival (DFS), overall survival (OS), distant metastasis-free survival (DMFS) and locoregional relapse-free survival (LRRFS) for EBV-negative group vs. EBV-positive group were 88.9% vs. 76.9% (*P* < 0.001), 93.6% vs. 85.9% (*P* = 0.001), 96.7% vs. 84.8% (*P* < 0.001) and 94.1% vs. 90.0% (*P* = 0.1), respectively. Multivariate analysis revealed that the EBV status was an independent prognostic factor for DFS (HR, 1.813; 95% CI, 1.219-2.695; *P* = 0.003), OS (HR, 1.828; 95% CI, 1.075-3.107; *P* = 0.026) and DMFS (HR, 3.678; 95% CI, 1.859-7.277; *P* <0.001), and overall stage still remained the most important prognostic factor in patients with stage III-IVB NPC.

**Methods and Materials:**

Data on 1106 patients with non-metastatic, histologically proven advanced-stage (III-IVB) NPC who underwent intensity-modulated radiotherapy (IMRT) were retrospectively reviewed. Patient survival between different EBV status groups were compared.

**Conclusions:**

EBV status was an independent prognostic factor for patients with stage III–IVB NPC. Neoadjuvant chemotherapy (NCT) plus concurrent chemoradiotherapy (CCRT) should be better treatment regimen for EBV-positive patients since distant metastasis was the main failure pattern, and CCRT may be enough for EBV-negative patients.

## INTRODUCTION

Worldwide, there are an estimated 84,400 new cases of nasopharyngeal carcinoma (NPC) and 51,600 NPC-related deaths in 2011 [1]. NPC has an obviously skewed geographical distribution, with an age-standardized incidence rate of 20–50 per 100 000 males in South China and a rate of 0.5 per 100 000 males in Caucasian populations [1]. Due to the anatomic constraints and its high degree of radiosensitivity, radiotherapy is the primary and only curative treatment for NPC. Despite its known limitations, TNM staging remains the most important prognostic factor in NPC currently [2]. Many new prognostic factors have also been reported, including plasma Epstein-Barr virus (EBV) DNA [3-5], primary tumor volume [6, 7], pre-treatment serum lactate dehydrogenase [8] and presence of comorbidities [9].

Since it was first reported by Lin et al. [3], the prognostic value of plasma EBV DNA has been widely investigated during the last decade. Recently, plasma EBV DNA has been used in the clinical setting [4, 10, 11] and has been established as a reliable biomarker for detection, monitoring and prognostic prediction in NPC patients [5, 12]. However, these previous studies were focusing on the prognostic value of pre-treatment plasma EBV DNA level in NPC patients with stage I–IV cancer, and no study has investigated the prognostic impact of pre-treatment EBV status in advanced stage NPC.

A recent review by Chua et al. showed that patients with non-viral-associated NPC had different pathogenesis and outcomes compared with patients with viral-associated tumors [13]. Moreover, patients with different EBV status may get different treatment strategies based on plasma EBV DNA level since the tumor burden could be represented by the plasma EBV DNA level [14]. Therefore, the prognostic difference between EBV-negative and EBV-positive patients is worth investigating.

On the basis of this premise, we conducted this retrospective study to evaluate the impact of tumor EBV status on survival outcomes in advanced-stage NPC patients in the era of intensity-modulated radiation therapy (IMRT).

## RESULTS

### Patient characteristics

Of the whole cohort, 248 (22.4%) patients with undetectable plasma pre-treatment EBV DNA were classified as the EBV-negative group, and the remaining were classified as the EBV-positive group. The male (841)-to-female (265) ratio was 3.2:1, and the median age was 45 years (range, 14–78 years). EBV-negative and EBV-positive patients were similar in terms of the majority of the host factors and histological categories. Additionally, the number of patients receiving pre-treatment PET-CT test in EBV-negative group was similar to that in EBV-positive group. However, a higher number of patients in the EBV-positive group than in the EBV-negative group had tumors classified as T4 (*P* < 0.001), N2-3 (*P* < 0.001) and stage IV (*P* < 0.001). Moreover, a higher percentage of Patients in the EBV-negative group than in EBV-positive group received IMRT alone (*P* < 0.001) (Table 1).

**Table 1:** Baseline Characteristics of the 1106 patients with Advanced-Stage NPC

Characteristics	EBV −	EBV +	*P*^a^
	No. (%)	No. (%)	
Gender			0.439
Male	184 (74.2)	657 (76.6)	
Female	64 (25.8)	201 (23.4)	
Age (years)			0.142
≥50	74 (29.8)	299 (34.8)	
<50	174 (70.2)	559 (65.2)	
WHO pathological classification			0.696
Type I	2 (0.8)	5 (0.6)	
Type II/III	246 (99.2)	853 (99.4)	
Family history			0.89
Yes	67 (27.0)	228 (26.6)	
No	181 (73.0)	630 (73.4)	
Smoking			0.288
Yes	91 (36.7)	347 (40.4)	
No	157 (63.3)	511 (59.6)	
Drinking			0.774
Yes	35 (14.1)	115 (13.4)	
No	213 (85.9)	743 (86.6)	
PET-CT			0.385
Yes	72 (29.0)	274 (31.9)	
No	176 (71.0)	584 (68.1)	
T classification^b^			<0.001
T1	13 (5.2)	37 (4.3)	
T2	7 (2.8)	53 (6.2)	
T3	193 (77.8)	523 (61.0)	
T4	35 (14.2)	245 (28.5)	
N classification^b^			<0.001
N0	68 (27.4)	53 (6.2)	
N1	137 (55.3)	474 (55.2)	
N2	34 (13.7)	203 (23.7)	
N3	9 (3.6)	128 (14.9)	
Overall stage^b^			<0.001
III	205 (82.7)	509 (59.3)	
IVA−IVB	43 (17.3)	349 (40.7)	
Treatment regimen			<0.001
IMRT	32 (12.9)	36 (4.2)	
IMRT + NCT/ACT	20 (8.1)	105 (12.2)	
CCRT +/− NCT/ACT	196 (79.0)	717 (83.6)	

Abbreviations: -, negative; +, positive; EBV = Epstein-Barr virus; WHO = World Health Organization; PET = positron emission tomography; NPC = nasopharyngeal carcinoma; IMRT = intensity-modulated radiotherapy; NCT = neoadjuvant chemotherapy; ACT = adjuvant chemotherapy; CCRT = concurrent chemoradiotherapy.

a *P* values were calculated using the chi-square test or Fisher exact test if indicated.

b According to the 7^th^ edition of the AJCC/UICC staging system.

### Failure patterns and treatment outcomes

The outcomes of treatment failure are summarized in Table 2. The median follow-up time for this entire cohort was 49.8 months (range, 1.3–70.7 months), and 198 (17.9%) patients were lost to follow-up. By the last follow-up, 11 (4.4%) patients in the EBV-negative group and 53 (6.2%) patients in the EBV-positive group developed local failure, 7 (2.8%) patients in the EBV-negative group and 40 (4.7%) patients in the EBV-positive group suffered regional failure, and 9 (3.6%) patients in the EBV-negative group and 129 (15.0%) patients in the EBV-positive group had distant failure. There was no significant difference between the two groups with regard to locoregional failure (*P* = 0.143). However, the percentage of distant metastasis events was significantly higher in the EBV-positive group (15.0% vs. 3.6%, *P* < 0.001) than that in the EBV-negative group. Moreover, there were 16 (6.5%) deaths in EBV-negative group and 125 (14.6%) deaths in EBV-positive group; the EBV-positive group had a higher percentage of cancer-related deaths (*P* = 0.005).

**Table 2:** Failure Patterns in EBV-negative and EBV-positive Patients with NPC

Failure patterns	EBV −(%)	EBV + (%)	P^a^
Local only	8 (3.2)	33 (3.8)	0.417
Local + regional	2 (0.8)	6 (0.7)	1.000
Local + distant	1 (0.4)	8 (0.9)	0.662
Local + regional + distant	0 (0)	6 (0.7)	0.407
Regional only	4 (1.6)	19 (2.2)	0.398
Regional + distant	1 (0.4)	9 (1.0)	0.557
Distant only	7 (2.8)	106 (12.4)	<0.001
Total locoregional	16 (6.5)	81 (9.4)	0.143
Total distant	9 (3.6)	129 (15.0)	<0.001
Total	23 (9.3)	187 (21.8)	<0.001
Cause of death			0.005
Cancer	10 (62.5)	111 (88.8)	
Non-cancer	6 (37.5)	14 (11.2)	
Total	16	125	

Abbreviations: -, negative; +, positive; EBV = Epstein-Barr virus; NPC = nasopharyngeal carcinoma.

a P values were calculated using the chi-square test or Fisher exact test if indicated.

### Univariate analysis

**Figure 1:**
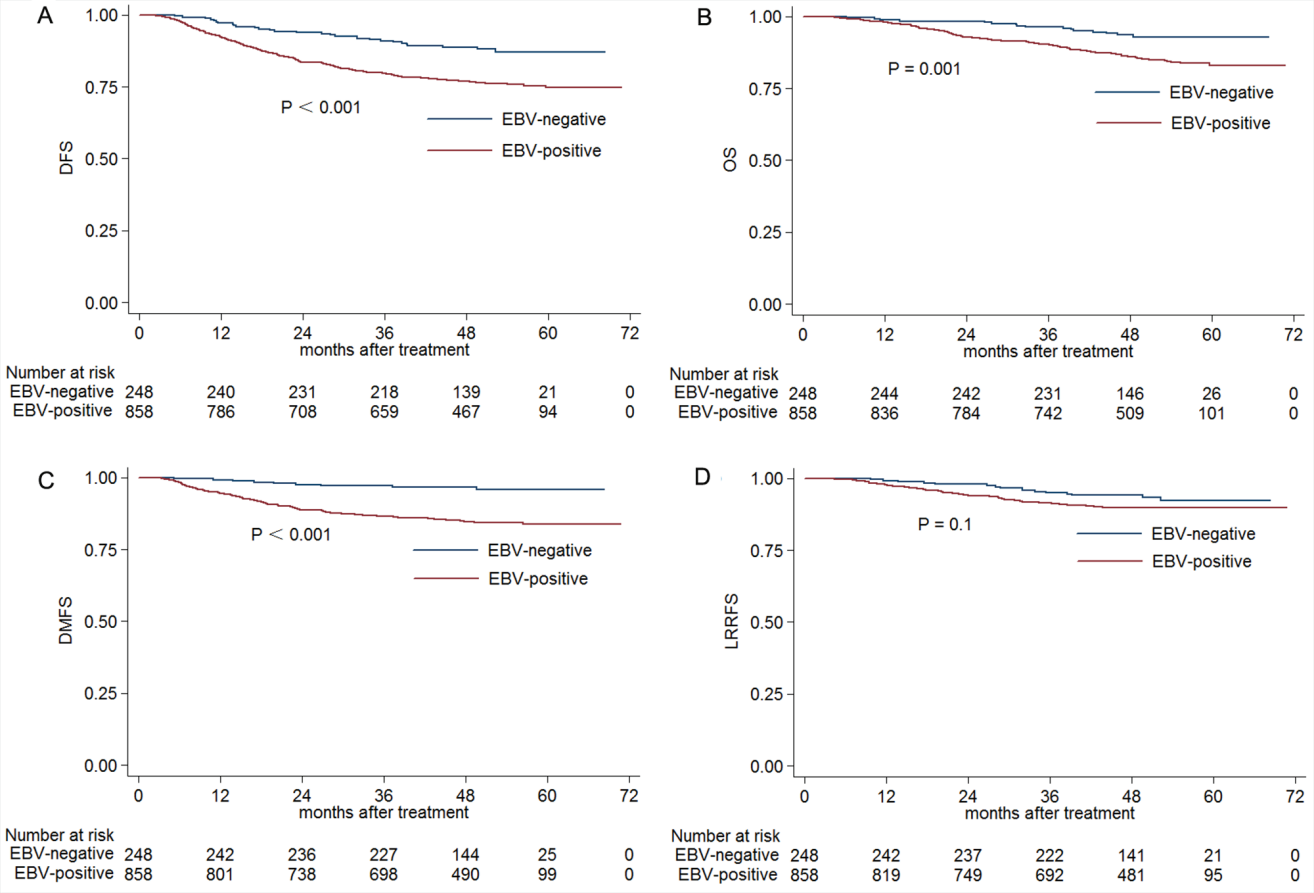
Kaplan-Meier DFS **A.** OS **B.** DMFS **C.** and LRRFS **D.** curves for NPC patients with stage III–IVB disease classified as EBV-negative and EBV-positive groups. Abbreviations: DFS = disease-free survival; OS = overall survival; DMFS = distant metastasis-free survival; LRRFS = local-regional relapse-free survival.

For the entire cohort, the estimated 4-year disease-free survival (DFS), overall survival (OS), distant metastasis-survival (DMFS) and locoregional relapse-free survival (LRRFS) were 79.6%, 87.6%, 87.4% and 90.9%, respectively. The estimated 4-year DFS, OS, DMFS and LRRFS rates for EBV-negative group vs. EBV-positive group were 88.9% vs. 76.9% (*P* < 0.001), 93.6% vs. 85.9% (*P* = 0.001), 96.7% vs. 84.8% (*P* < 0.001) and 94.1% vs. 90.0% (*P* = 0.1) (Figure 1), respectively.

### Multivariate analysis

Multivariate analysis was performed to adjust for various prognostic factors. The findings from multivariate analysis are listed in Table 3. EBV status was found to be an independent prognostic factor for poor DFS (HR, 1.813; 95% CI, 1.219–2.695; *P* = 0.003), OS (HR, 1.828; 95% CI, 1.075–3.107; *P* = 0.026), and DMFS (HR, 3.678; 95% CI, 1.859–7.277; *P* < 0.01). However, EBV-positive patients with NPC had the similar risk for locoregional failure as EBV-negative patients (HR, 1.452; 95% CI, 0.838–2.516; *P* = 0.184). Of note, overall stage had better prognostic value in DFS and OS compared with EBV status (Table 3).

**Table 3:** Multivariate Analysis of Patients with Stage III–IV NPC

Endpoint	Variable	*P*^a^	HR	95% CI for HR
DFS	EBV status	0.003	1.813	1.219–2.695
	Age	0.011	1.409	1.083–1.832
	Overall stage	< 0.001	1.850	1.421-2.410
OS	EBV status	0.026	1.828	1.075–3.107
	Age	<0.001	1.881	1.349–2.623
	Pathology	0.049	0.315	0.1–0.997
	Overall stage	<0.001	2.273	1.613-3.203
DMFS	EBV status	<0.001	3.678	1.859–7.277
	Overall stage	<0.001	2.022	1.441–2.836
LRRFS	Pathology	0.002	0.165	0.052–0.521
	Overall stage	0.02	1.610	1.078-2.403

Abbreviations: EBV = Epstein-Barr virus; NPC = nasopharyngeal carcinoma; DFS = disease-free survival; OS = overall survival; DMFS = distant metastases-free survival; LRRFS = loco-regional relapse-free survival; HR = hazard ratio; CI = confidence interval.

a P values were calculated using an adjusted Cox proportional hazards model.

The following variables were included in the Cox proportional hazards model with backward elimination: gender (male vs. female), age (≥50 y vs. <50 y), pathology (type I vs. type II/III), family history (yes vs. no), smoking (yes vs. no), drinking (yes vs. no), overall stage (III vs. IV), treatment regimen (IMRT vs. IMRT + NCT/ACT vs. CCRT +/− NCT/ACT), EBV status (negative vs. positive).

### Subgroup analysis according to the clinical stage

To further investigate the prognostic value of the EBV status for different clinical tumor stages, subgroup analysis was conducted according to the T, N and overall stage (Table 4). The results of the subgroup analysis showed that the prognostic differences between the EBV-negative and EBV-positive groups were prominent in patients with stage T3, N1 and III cancer. Multivariate analysis showed that the EBV status was an independent prognostic factor for DMFS (HR, 2.933; 95% CI, 1.251–6.877; *P* = 0.013) in the stage T3 subgroup; for DFS (HR, 1.696; 95% CI, 1.006–2.859; *P* = 0.047), OS (HR, 2.266; 95% CI, 1.027–4.999; *P* = 0.043) and DMFS (HR, 4.980; 95% CI, 1.554–15.955; *P* = 0.007) in the stage N1 subgroup; for DFS (HR, 1.849; 95% CI, 1.151–2.972; *P* = 0.011), OS (HR, 2.798; 95% CI, 1.314–5.954; *P* = 0.008) and DMFS (HR, 3.984; 95% CI, 1.718–9.239; *P* = 0.001) in the stage III subgroup.

**Table 4:** Subgroup Analysis of the EBV-negative and EBV-positive Groups According to the Clinical Stage

	4-year DFS			4-year OS			4-year DMFS			4-year LRRFS		
Stage	EBV –	EBV +	*P*^a^	EBV -	EBV +	*P*^a^	EBV -	EBV +	*P*^a^	EBV -	EBV +	*P*^a^
	(%)	(%)		(%)	(%)		(%)	(%)		(%)	(%)	
T												
T1	84.6	64.3	0.211	92.3	82.7	0.466	100	74.1	0.063	91.7	90.9	0.952
T2	85.7	75.1	0.616	83.3	82.6	0.882	85.7	84.4	0.994	100	89.7	0.409
T3	90.5	81.0	0.004	96.2	89.2	0.005	97.3	87.6	0.001	94.1	92.3	0.67
T4	82.4	70.5	0.121	81.2	80.2	0.575	94.2	80.4	0.061	94.2	85.0	0.165
N												
N0	92.6	84.7	0.159	93.3	90.3	0.437	97.1	92.4	0.241	95.6	92.0	0.415
N1	88.0	81.2	0.065	94.7	89.1	0.03	97.8	88.8	0.002	92.4	90.9	0.714
N2	87.7	72.5	0.102	90.9	83.3	0.269	93.6	81.2	0.148	96.7	87.1	0.274
N3	76.2	64.7	0.539	88.9	76.6	0.895	85.7	72.1	0.39	100	90.1	0.382
Overall												
III	90.5	82.1	0.009	96.0	89.9	0.007	97.5	88.9	<0.001	94.0	91.9	0.602
IV	80.8	69.3	0.113	82.2	80.1	0.692	92.7	78.6	0.044	95.1	87.0	0.151

Abbreviations: EBV = Epstein-Barr virus; DFS = disease-free survival; OS = overall survival; DMFS = distant metastases-free survival; LRRFS = loco-regional relapse-free survival. -, negative; +, positive.

a P values were calculated using the unadjusted log-rank test.

## DISCUSSION

To the best of our knowledge, this current study is the first large-scale study to report the impact of EBV status on the prognosis of patients with stage III–IVB NPC in a population with a high prevalence of EBV infection and NPC. Distant metastasis had been the main treatment failure pattern for EBV-positive patients. The results of univariate analysis may be less meaning because of the unbalanced distribution of tumor stage and treatment intensity. However, the outcomes of multivariate analysis showed that patients with positive EBV have an obviously poorer prognosis than EBV-negative patients, and overall stage still remained the most important prognostic factor. Moreover, subgroup analysis revealed that this difference was mainly observed in patients with T3, N1 disease and overall stage III.

Previous study had discussed the different pathogenesis in EBV-negative and EBV-positive NPC [13]. EBV-associated NPC follows a stepwise malignant transformation consists of latent EBV infection, evasion of host immune surveillance, loss of heterogeneity at specific chromosomal regions, genetic mutations and activation of oncogenic signaling pathways, and epigenetic silencing of tumor suppressor genes [15, 16]. However, human papilloma virus (HPV) has been considered as a contributing factor in EBV-negative NPC in non-endemic regions [17-21] and is correlated to poor prognosis [21]. However, patients with negative EBV in our study had obviously better prognosis than EBV-positive patients, which was inconsistent with the results of the study by Stenmark et al. [21]. This indicated that EBV-negative patients in the endemic region did not have HPV infection.

Of note, a higher percentage of patients in the EBV-positive group had advanced-stage NPC than patients in the EBV-negative group; which indicating that EBV-positive patients had a greater tumor burden than EBV-negative patients. Therefore, different treatment strategies should be performed for EBV-negative and EBV-positive patients before, during and after treatment. In pre-treatment phase, thorough evaluation including PET-CT examination should be performed for EBV-positive patients to enhance the accuracy of tumor stage since patients with positive EBV had a higher percentage of advanced-stage NPC compared with EBV-negative patients. The outcomes of this current study revealed that distant metastasis was the main failure pattern for EBV-positive patients. Therefore, in treatment phase, neoadjuvant chemotherapy (NCT) plus concurrent chemoradiotherapy (CCRT) may be better treatment regimen for EBV-positive patients, and CCRT may be enough for EBV-negative patients since distant failure was not the main failure pattern. Previous studies reported that recurrence or distant metastases could be detected in time based on the post-treatment plasma EBV DNA level [22, 23]. In the follow-up phase, clinicians should therefore gain more insight into the follow-up of EBV-negative patients since the disease recurrence could not be detected based on post-treatment plasma EBV DNA level. Moreover, post-treatment EBV DNA should be frequently detected since distant metastasis is the main treatment failure for EBV-positive patients.

In the subgroup analysis, the difference in prognosis was mainly noted in the subgroups T3, N1 and III. For patients with stage T4 or N2-3 NPC, the tumor burden and risk of distant metastases were high in both the EBV-negative and EBV-positive groups. Therefore, the poor prognosis in patients with advanced stage NPC may dilute the prognostic impact of EBV. For patients with T1-2 and N0 disease, the tumor burden was small, and EBV-positive patients had a very low plasma EBV DNA level [5, 23, 24]. Hence, the prognostic impact of EBV may be not obvious. Hence, in clinical practice, patients with stage T3, N1 and III stage and positive plasma EBV DNA may need more intensive chemotherapy regimens like NCT + CCRT. However, CCRT alone may be enough for patients with negative plasma EBV DNA and stage T3, N1 and III disease.

The current study revealed that patients who are negative for EBV have a better prognosis than those who are positive for EBV. However, two main limitations may exist in this current study. First, our study was a retrospective one for which the follow-up time was insufficient, so DFS was chosen as the major endpoint to address this shortcoming. Moreover, the judgement of EBV status was only based on plasma EBV DNA level and more accurate diagnosed criteria should be used in future studies. Future prospective clinical studies should be warranted to confirm the results of this current study.

## MATERIALS AND METHODS

### Patient selection

We retrospectively analysed 1811 patients with previously untreated, biopsy-proven NPC with no evidence of distant metastasis, who were treated between November 2009 and February 2012 at Sun Yat-sen University Cancer Center. Patients with advanced-stage NPC (III–IVB) and pre-treatment plasma EBV DNA data were recruited, so only 1106 patients were analysed further. This study was conducted in compliance with the institutional policy regarding the protection of patients' private information and was approved by the Research Ethics Committee of Sun Yat-sen University Cancer Center. Informed consent was obtained from all the patients.

### Clinical staging

The routine staging workup included a complete history and clinical examinations of the head and neck region, direct fibre-optic nasopharyngoscopy, magnetic resonance imaging (MRI) of the skull base and whole neck, chest radiography, whole-body bone scan and abdominal sonography, as well as positron emission tomography (PET)-CT if clinical indicated. Tumour-related plasma EBV DNA load was tested. All patients underwent a dental evaluation before radiotherapy.

All patients were restaged according to the 7^th^ edition of the International Union against Cancer/American Joint Committee on Cancer (UICC/AJCC) system [25]. All MRI materials and clinical records were reviewed to minimize heterogeneity in the restaging. Two radiologists (L.Z.L. and L.T.) employed at our hospital separately evaluated all the scans, and disagreements were resolved by consensus.

### DNA extraction and real-time quantitative PCR

Before treatment, peripheral blood (3 ml) was collected from each patient, placed in an ethylene diamine tetra acetic-coated tube, and centrifuged at 1600 *g* for 15 min to isolate plasma and peripheral blood cells (PBCs). The plasma samples were stored at −80°C until further processing. DNA was extracted from plasma using the QIAamp Blood Kit (Qiagen, Hilden, Germany) and the blood and body fluid protocol recommended by the manufacturer. A total of 500 μl of each plasma sample was used for DNA extraction per column, and the final elution volume was 50 μl per column.

The concentration of EBV DNA in the plasma was measured using a real-time quantitative PCR assay targeting the *BamH* I-W region of the EBV genome. The sequences of the forward and reverse primers were 5′-GCCAG AGGTA AGTGG ACTTT-′ and 5′-TACCA CCTCC TCTTC TTGCT-3′ respectively. A dual fluorescence-labelled oligomer, 5′-(FAM)CACAC CCAGG CACAC ACTAC ACAT(TAMRA)-3′, served as the probe. Sequence data for the EBV genome were obtained from the GenBank sequence database. The principles of the real-time quantitative PCR assay and detailed reaction setup procedures were as described previously [4, 24]. The plasma EBV DNA concentration was calculated using the following equation: *C = Q × (V_DNA_*/V*_PCR_*) ×(1/V*_EXT_*), in which *C* represents the target concentration in plasma (copies/ml), *Q* represents the target quantity (copy number) determined by PCR, *V_DNA_* represents the total volume of DNA obtained after extraction (typically 50 μl/Qiagen extraction), *V_PCR_* represents the volume of DNA solution used for PCR (typically 2 μl), and *VEXT* represents the volume of plasma extracted (typically 0.5 ml) [24].

## CLINICAL TREATMENT

### Radiotherapy

All patients underwent IMRT at Sun Yat-sen University Cancer Center. Immobilization was carried out using a custom-made head-to neck-thermoplastic cast with the patient's neck resting on a support. A high-resolution planning CT scan with contrast was taken from the vertex to 2 cm below the sternoclavicular joint at a slice thickness of 3 mm. Target volumes were delineated slice-by-slice on treatment planning CT scans using an individualized delineation protocol that complies with the guidelines of the International Commission on Radiation Units and Measurements reports 50 and 62. The prescribed doses were 66–72 Gy at 2.12–2.43 Gy/fraction to the planning target volume (PTV) of the primary gross tumour volume (GTVnx), 64–70 Gy to the PTV of the GTV of the involved lymph nodes (GTVnd), 60–63 Gy to the PTV of the high-risk clinical target volume (CTV1), and 54–56 Gy to the PTV of the low-risk clinical target volume (CTV2). All targets were treated simultaneously using the simultaneous integrated boost technique.

### Chemotherapy

According to our institutional guidelines, before commencing treatment, we recommended radiotherapy alone for stage I disease, concurrent chemoradiotherapy for stage II disease, and concurrent chemoradiotherapy (CCRT) +/− neoadjuvant/adjuvant chemotherapy for stage III to IVB disease. Neoadjuvant or adjuvant chemotherapy consisted of cisplatin with 5-fluorouracil, cisplatin with taxels (docetaxel and paclitaxel) or a triplet of cisplatin and 5-fluorouracil plus taxels every three weeks for two or three cycles. Concurrent chemotherapy consisted of weekly cisplatin (30-40 mg/m^2^) or 3-weekly cisplatin (80-100 mg/m^2^) on weeks 1, 4 and 7 of radiotherapy.

### Follow-up and statistical analysis

Patient follow-up was conducted from the first day of therapy to the day of last examination or death. Patients were examined at least every 3 months during the first 2 years, with follow-up examinations conducted every 6 months thereafter until death. DFS was set as the first endpoint, and secondary endpoints included OS, DMFS and LRRFS.

The chi-square test or Fisher exact test was used to compare categorical variables and treatment failure patterns between the EBV-positive and EBV-negative group. Life-table estimation was performed using the Kaplan-Meier method, and differences were compared using the log-rank test. The multivariate Cox proportional hazards model was used to estimate the hazard ratios (HRs) and calculate 95% confidence intervals (CIs). The variables input in the model included age, gender, pathology type, T classification, N classification, chemotherapy, pre-treatment EBV DNA level, smoking and drinking. All statistical tests were two-sided; *P* < 0.05 was considered to indicate statistical significance. Stata Statistical Package (STATA 12; StataCorp LP, College Station, TX, USA) was used for all the analyses.

## CONCLUSION

In summary, our current study confirmed that the EBV status was found to be an independent prognostic factor for patients with stage III–IVB NPC. NCT plus CCRT should be better treatment regimen for patients with positive EBV since distant metastasis was the main failure pattern, and CCRT may be enough for EBV-negative patients since distant metastasis was not the main failure pattern.

## SUPPLEMENTARY METHODS TABLES


