# Supplementary material for: Survival analysis of patients with advanced-stage nasopharyngeal carcinoma according to the Epstein-Barr virus status
Source: Oncotarget. 2016 Mar 17;7(17):24208–16. doi: 10.18632/oncotarget.8144 (PMC5029695; doi:10.18632/oncotarget.8144)
Supplement: Supplementary file 1 [file oncotarget-07-24208-s001.pdf]

SUPPLEMENTARY METHODS

In order to control the heterogeneity, we conducted the subgroup analysis based on different treatment regimens to analyze the prognostic value of plasma EBV status. For the 68 (6.2%) patients receiving IMRT alone, multivariate analysis was conducted to investigate the prognostic value of EBV status (Supplementary Table S1). The results of multivariate analysis revealed that only overall stage was an independent prognostic factor for DFS and OS. The reasonable explanation may be the small sample and events in this subgroup. For the 125 (11.3%) patients receiving IMRT +/- NCT/ACT, the outcomes of multivariate analysis (Supplementary Table S2) was similar to the results above. The relatively small sample may also contribute to these results. For the 913 (82.5%) patients receiving CCRT +/- NCT/ACT, the results of multivariate analysis showed that EBV status was an independent prognostic factor for DFS and DMFS (Supplementary Table S3). Because the standard treatment regimen for advanced-stage NPC is CCRT +/- NCT/ACT and most patients in this study received the standard treatment, we therefore could conclude that EBV status is an independent prognostic factor in advanced NPC. Of note, the overall stage had been the most important prognostic factor after replacing T-classification and N-classification with overall stage, and the prognostic value of EBV status in OS may be covered by it. Therefore, EBV status was found to have no prognostic value in OS after replacing T-classification and N-classification with overall stage.

SUPPLEMENTARY TABLES

Supplementary Table S1: Multivariate Analysis of Patients with Stage III–IV NPC Receiving IMRT alone

Endpoint	Variable	P^a	HR	95% CI for HR
DFS	Overall stage	0.048	3.604	1.011-12.852
OS	Overall stage	0.01	14.756	1.928-112.928

Abbreviations: NPC = nasopharyngeal carcinoma; DFS = disease-free survival; OS = overall survival; HR = hazard ratio; CI = confidence interval; IMRT = intensity-modulated radiotherapy.
[a]P values were calculated using an adjusted Cox proportional hazards model.
The following variables were included in the Cox proportional hazards model with backward elimination: gender (male vs. female), age (≥50 y vs. <50 y), pathology (type I vs. type II/III), family history (yes vs. no), smoking (yes vs. no), drinking (yes vs. no), Overall stage (III vs. IV), EBV status (negative vs. positive).

Supplementary Table S2: Multivariate Analysis of Patients with Stage III–IV NPC Receiving IMRT + NCT/ACT

Endpoint	Variable	P^a	HR	95% CI for HR
DFS	Pathology	0.026	0.089	0.011-0.746
	Overall stage	0.005	3.253	1.419-7.457
OS	Overall stage	0.013	4.443	1.378-14.326

Abbreviations: NPC = nasopharyngeal carcinoma; DFS = disease-free survival; OS = overall survival; HR = hazard ratio; CI = confidence interval; IMRT = intensity-modulated radiotherapy; NCT = neoadjuvant chemotherapy; ACT = adjuvant chemotherapy.
[a]P values were calculated using an adjusted Cox proportional hazards model.
The following variables were included in the Cox proportional hazards model with backward elimination: gender (male vs. female), age (≥50 y vs. <50 y), pathology (type I vs. type II/III), family history (yes vs. no), smoking (yes vs. no), drinking (yes vs. no), Overall stage (III vs. IV), EBV status (negative vs. positive).

Supplementary Table S3: Multivariate Analysis of Patients with Stage III–IV NPC Receiving CCRT +/- NCT/ACT

Endpoint	Variable	P^a	HR	95% CI
DFS	EBV status	0.006	1.892	1.204-2.972
	Overall stage	< 0.001	1.742	1.300-2.334
OS	Age	0.001	1.919	1.324-2.781
	Overall stage	< 0.001	2.118	1.450-3.092
DMFS	EBV status	0.003	2.886	1.452-5.738
	Overall stage	< 0.001	1.984	1.370-2.873
LRRFS	Pathology	0.023	0.195	0.048-0.796

Abbreviations: NPC = nasopharyngeal carcinoma; DFS = disease-free survival; OS = overall survival; HR = hazard ratio; DMFS = distant metastasis-free survival; LRRFS = locoregional relapse-free survival; CI = confidence interval; CCRT = concurrent chemoradiotherapy; NCT = neoadjuvant chemotherapy; ACT = adjuvant chemotherapy.
[a]P values were calculated using an adjusted Cox proportional hazards model.
The following variables were included in the Cox proportional hazards model with backward elimination: gender (male vs. female), age (≥50 y vs. <50 y), pathology (type I vs. type II/III), family history (yes vs. no), smoking (yes vs. no), drinking (yes vs. no), Overall stage (III vs. IV), EBV status (negative vs. positive).
